# Prion Infected Meat-and-Bone Meal Is Still Infectious after Biodiesel Production

**DOI:** 10.1371/journal.pone.0002969

**Published:** 2008-08-13

**Authors:** Cathrin E. Bruederle, Robert M. Hnasko, Thomas Kraemer, Rafael A. Garcia, Michael J. Haas, William N. Marmer, John Mark Carter

**Affiliations:** 1 USDA-ARS WRRC, Foodborne Contaminants Research Unit, Albany, California, United States of America; 2 Forensic Toxicology, Institute of Legal Medicine, Saarland University, Homburg/Saar, Germany; 3 USDA-ARS ERRC, Fats, Oils and Animal Coproducts Research Unit, Wyndmoor, Pennsylvania, United States of America; University of Edinburgh, United Kingdom

## Abstract

The epidemic of bovine spongiform encephalopathy (BSE) has led to a world-wide drop in the market for beef by-products, such as Meat-and-Bone Meal (MBM), a fat-containing but mainly proteinaceaous product traditionally used as an animal feed supplement. While normal rendering is insufficient, the production of biodiesel from MBM has been suggested to destroy infectivity from transmissible spongiform encephalopathies (TSEs). In addition to producing fuel, this method simultaneously generates a nutritious solid residue. In our study we produced biodiesel from MBM under defined conditions using a modified form of alkaline methanolysis. We evaluated the presence of prion in the three resulting phases of the biodiesel reaction (Biodiesel, Glycerol and Solid Residue) in vitro and in vivo. Analysis of the reaction products from 263K scrapie infected MBM led to no detectable immunoreactivity by Western Blot. Importantly, and in contrast to the biochemical results the solid MBM residue from the reaction retained infectivity when tested in an animal bioassay. Histochemical analysis of hamster brains inoculated with the solid residue showed typical spongiform degeneration and vacuolation. Re-inoculation of these brains into a new cohort of hamsters led to onset of clinical scrapie symptoms within 75 days, suggesting that the specific infectivity of the prion protein was not changed during the biodiesel process. The biodiesel reaction cannot be considered a viable prion decontamination method for MBM, although we observed increased survival time of hamsters and reduced infectivity greater than 6 log orders in the solid MBM residue. Furthermore, results from our study compare for the first time prion detection by Western Blot versus an infectivity bioassay for analysis of biodiesel reaction products. We could show that biochemical analysis alone is insufficient for detection of prion infectivity after a biodiesel process.

## Introduction

Bovine spongiform encephalopathy (BSE) belongs to the transmissible spongiform encephalopathies (TSEs), a family of fatal neurodegenerative diseases. Besides BSE in cows (‘mad cow disease’), other TSEs include Chronic Wasting Disease (CWD) in elk and deer, Creutzfeldt-Jacob Disease (CJD) in humans and scrapie in sheep.

The hallmark of TSEs is the conversion of cellular prion protein (PrP^c^) to an abnormal isoform (prion, PrP^sc^) relatively rich in beta sheet structure [1]. It is generally recognized that PrP^sc^ itself is the infectious agent responsible for onset of disease [2]. Both PrP isoforms are encoded by the same gene, exhibit the same amino acid sequence but differ in conformation [3].

Insolubility and protease resistance is used to distinguish PrP^sc^ from PrP^c^ biochemically. Limited proteolysis of PrP^sc^ by proteinase K but not PrP^c^ produces a smaller protease resistant residue of approximately 142 amino acids that can be detected by Western Blot [4]. Importantly, this molecular weight difference can only be used as surrogate marker. The absence of the described characteristics does not exclude infectivity.

Unlike viruses, prions are infectious proteins and transmissibility is not dependent on nucleic acid [5]. Furthermore, prions are remarkably resistant to standard decontamination procedures that are usually used to degrade viruses and bacteria including irradiation, boiling, dry heat and most chemicals. Decontamination of prions is especially difficult and expensive and poses challenging problems in a large scale format such as would be required in slaughterhouses. Inactivation of prions can be achieved by steam autoclaving at temperatures greater than 121°C and prior treatment with 1M NaOH, or by boiling in 1M NaOH [6], [7], techniques not suitable for large amounts of material.

Another defining characteristic of TSEs is transmissibility between individuals or across species [8], [9]. Inter-species transmission with unintentional application of incomplete inactivation procedures is thought to be responsible for the outbreak of the BSE epidemic in Europe. Specifically, BSE has been traced to Meat-and-Bone Meal (MBM) derived from scrapie contaminated sheep offal. [10]. MBM, a high protein meal, is produced through rendering of animal carcasses after the valuable tissue parts have been harvested in slaughterhouses [11]. MBM has been traditionally used as an animal feed supplement, but the use has been suspended in Europe due to food safety and health concerns for consumers [12], [13]. In the United States, where the incidence of BSE has been very low, ruminant-derived MBM is only used as animal feed for non-ruminants [14]. This raises the question how to better utilize beef coproducts such as MBM that accumulate in huge amounts during the processing of cattle in slaughterhouses.

Production of biodiesel from animal fat has been proposed to decontaminate any potential prion infectivity present in the biodiesel [15]. Production of biodiesel from MBM can produce a valuable fuel and a nutritious solid MBM residue that could be used as feed additive. The production and use of fatty acid methyl esters (FAME) as a fuel for diesel engines is a rapidly growing technology that has penetrated the transportation fuels sector in Europe and is in the process of doing so in the U.S. and other countries. Compared to petroleum-derived diesel fuel biodiesel offers advantages such as reduced emissions, domestic sourcing and renewability [16].

In our study we produced biodiesel from MBM spiked with 263K scrapie, a model for TSE infectivity, using *in situ* alkaline methanolysis with 0.25% sodium methoxide. This represents a low cost method suitable for biodiesel production from MBM. *In situ* transesterification has been described previously to be generally applicable for FAME synthesis from lipid-bearing materials such as MBM [17].

We validated biodiesel production from MBM by gas chromatography and evaluated all three phases derived from the reaction (Biodiesel, Glycerol and Solid MBM Residue) for the presence of proteinase K resistant prion biochemically and prion infectivity by animal bioassay.

## Results

### Low temperature alkaline methanolysis of MBM leads to efficient biodiesel production

**Figure 1:**
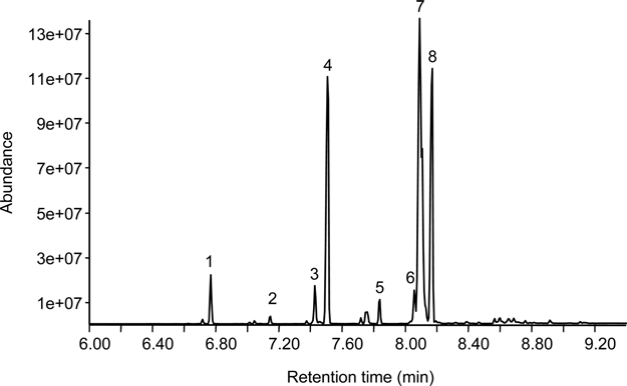
Low temperature alkaline methanolysis leads to efficient biodiesel production from MBM spiked with 5% hamster brain. After alkaline methanolysis of MBM the upper biodiesel phase was separated and analyzed by GC-MS. GC-MS results show the alkaline methanolysis reaction produced various fatty acid methyl esters (FAME) which are typical components of biodiesel (see Table 1 for FAME composition). The two most abundant fatty acid methyl esters were analyzed quantitatively by using internal deuterated standards and calibration curve (Table 2).

MBM entirely derived from cattle bones and offal was spiked with 5% brain homogenate from either normal or 263K scrapie infected hamsters, and then subjected to alkaline methanolysis. The reaction was performed by vigorous shaking of MBM in 0.25% sodium methoxide for 2 hours at 35°C. After settling of the mixture overnight we could distinguish 3 independent reaction phases. The upper phase, representing the potential biodiesel, was separated and analyzed by gas chromatography (Figure 1). Qualitative analysis revealed several fatty acid methyl esters that are common constituents of biodiesel (Table 1). Furthermore the biodiesel phase was analyzed quantitatively using internal deuterated standards (Table 2). Taken together these data show that we efficiently produced biodiesel with a FAME composition comparable to previous reported results [17]. Qualitative and quantitative analysis together suggest reasonable purity of the biodiesel derived from spiked MBM.

**Table 1:** Alkaline methanolysis reaction produced various fatty acid methyl esters which are typical components of biodiesel *

1	Tetradecanoic acid methyl ester
2	Pentadecanoic acid methyl ester
3	Hexadecenoic acid methyl ester
4	Hexadecanoic acid methyl ester
5	Heptadecanoic acid methyl ester
6	Octadecadienoic acid methyl ester
7	Octadecenoic acid methyl ester
8	Octadecanoic acid methyl ester

* Numbers indicate the spikes shown in Figure 1

**Table 2:** Quantification of the two most abundant fatty acid methyl esters using internal deuterated standards*

Fatty Acid Methyl Ester (FAME)	Percentage of total FAME produced
Stearate (Octadecanoic acid methyl ester)	20%
Palmitate (Hexadecanoic acid methyl ester)	17%

* calculated according to Haas et al.

### Inocula prepared from non infectious biodiesel fractions are not toxic

In order to validate the infectivity of the three different resulting phases from the biodiesel reaction we needed to perform an *in vivo* assay in Syrian hamsters that involved intracranial inoculation (*ic*) of the generated material [18]. First we evaluated biodiesel reaction fractions from MBM spiked with 5% uninfected brain (MBM c) for potential toxic effects in our animal bioassay. This was necessary to exclude death of animals due to acute toxicity from inoculation of biodiesel, glycerol or the solid MBM residue and not due to scrapie infectivity. The 3 reaction phases were diluted 1∶10^3^ and 1∶10^4^ into 320 mM sucrose and 50 µl of each sample was inoculated into Syrian hamsters (Figure S1 and Table S1). Animals were monitored closely over 72 hours. No acute toxic effects of the inocula were observed and all animals survived treatment. The study was terminated by sacrificing the hamsters after 90 days. We also analyzed the inocula (as diluted) via Western Blot. Interestingly we could not detect PrP in the inoculated phases, suggesting destruction of proteinaceaous material by alkaline methanolysis. Normal brain homogenate and MBM c served as positive control. PrP was clearly visible at the expected molecular weight in these control samples (Figure S1).

### Solid MBM residue from biodiesel reaction retains infectivity

In order to investigate the decontamination potential of the biodiesel reaction under our defined conditions we performed alkaline methanolysis on MBM spiked with 5% 263K scrapie infected brain (MBM sc). After the reaction the three chemical phases were separated and each phase was subsequently diluted to an estimated amount of 25 µg of total protein into 50 µl of 320 mM sucrose solution to prepare a homogeneous sample for intracranial (*ic*) inoculation. The calculation of total protein was based on the input of brain equivalent before reaction and the assumption that all proteinaceaous material partitioned into the respective phase. Samples were divided and either inoculated into Syrian hamsters or analyzed by Western Blot for presence of PrP^sc^.

We prepared an identical sample set performing alkaline methanolysis on MBM c as negative control for *in vitro* and *in vivo* experiments.

#### Western analysis

**Figure 2:**
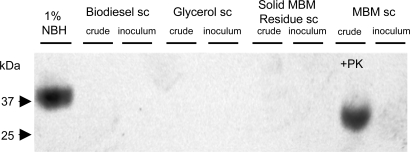
Western blot analysis of reaction products from MBM spiked with 5% scrapie brain. We performed alkaline methanolysis on scrapie brain spiked MBM as described under Methods and analyzed the reaction products as well as the inoculation samples by Western blot using monoclonal (mAb) 3F4. Results show no PrP detected in any of the 3 phases produced by the reaction (crude), nor in the inoculum we subsequently prepared by diluting each phase to 25 µg of total brain equivalent protein into 50 µl of 320 mM sucrose. After proteinase K digestion PrP^sc^ was clearly detected in MBM spiked with infected brain but not subjected to the reaction (MBM sc, crude +PK). 1% NBH served as a control, displaying a slightly higher molecular weight, since it was not proteinase K digested.

First we performed Western Blots on the crude fractions from the reaction as well as on the above described inocula (Figure 2). Western Blot results show no PrP detected in any of the 3 phases produced by the reaction (crude) or in the inoculum. After proteinase K digestion PrP^sc^ was clearly detected in MBM spiked with infected brain but not subjected to the reaction (MBM sc crude), as expected. Notable here is that we could not detect any protease resistant material in the corresponding inoculum at this dilution by Western Blot (MBM sc inoculum). 1% NBH served as a control, displaying a slightly higher molecular weight, since it was not digested by proteinase K (Figure 2). These data demonstrate that alkaline methanolysis decreased the amount of PrP^sc^ below the detection limit of Western Blot in the biodiesel, glycerol, and solid MBM residue phase.

#### Infectivity bioassay

**Figure 3:**
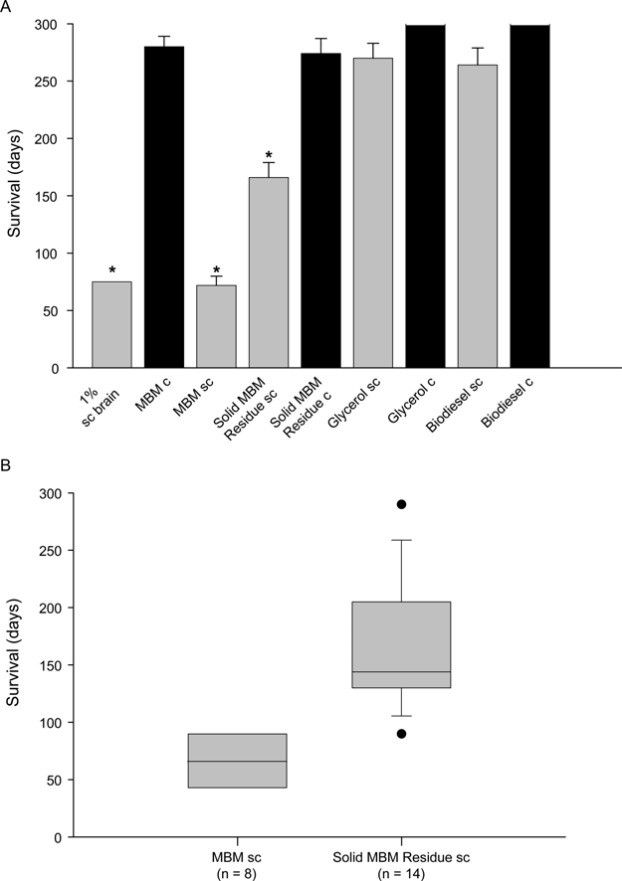
Infectivity bioassay. (A) Control animals inoculated with 50 µl 1% scrapie BH exhibited clinical symptoms 75 days after inoculation and were sacrificed (1% sc brain). Animals inoculated with MBM sc began to display clinical symptoms around 75 days post inoculation and were sacrificed when moribund. All animals inoculated with Solid MBM Residue sc developed disease (166+/−13 days), even though no PrP^sc^ was detected by Western Blot in initial inoculum. Survival time of animals from all 3 groups was significantly shorter as compared to control animals (control: MBM c and Solid MBM Residue c; p<0.05; ANOVA on ranks). Animals inoculated with Biodiesel sc and Glycerol sc displayed no clinical symptoms within the time frame of the experiment (>200 days). Survival was comparable to control group animals (Biodiesel c, Glycerol c). Days of survival are calculated as mean values. (B) The range of time-to death values of hamsters inoculated with MBM sc and Solid MBM Residue sc is summarized showing the median, upper and lower quartiles and extreme values (outliers) for comparison of both groups.

To confirm our results obtained by Western Blot we used a hamster bioassay to assess prion infectivity in all three reaction phases. Syrian hamsters were inoculated with biodiesel, glycerol or solid MBM residue produced from either MBM sc or uninfected MBM c (negative control). Furthermore, we inoculated untreated MBM c and MBM sc as pre-reaction controls. A group of hamsters inoculated with 1% scrapie brain homogenate served as additional control to prove the integrity of our animal assay (Table 3). These animals showed clinical scrapie symptoms around 65 days after inoculation and were sacrificed after 75 days, which is comparable to previous described results [19] Animals inoculated with a suspension of MBM sc began to display clinical symptoms around 75 days post inoculation and were sacrificed when moribund. Three animals from this group died after only 45 days, but death was apparently not due to scrapie, as the animals did not exhibit typical scrapie symptoms (Figure 3, Table 3).

**Table 3:** Solid MBM Residue remains infectious after alkaline methanolysis.

Inoculum	spiked with Infected Brain (Sacrificed Animals/Total animals)	Day Sacrificed
Biodiesel phase	4/14 **	**>200**
Glycerol phase	5/14 **	**>200**
**Solid MBM Residue+5% brain**	**14/14** ***	**166+/−13** *
**MBM+5% brain**	**8/8 ** ***	**72+/−8** *
**Brain Homogenate 1%**	**6/6 ** ***	**75**

* mean+/−standard error

** unsacrificed animals survived longer than 200 days, until study was terminated, without clinical scrapie symptoms

*** sacrificed animals exhibited clinical scrapie symptoms

Most interestingly, all animals inoculated with the solid MBM residue produced by the reaction with scrapie brain spiked MBM developed disease (Table 3: Solid MBM Residue +5% infected brain; Figure 3: Solid MBM Residue sc), even though no PrP^sc^ could be detected pre-inoculation by Western Blot (Figure 2: Solid MBM Residue sc). These results show that our biodiesel method failed to completely destroy infectivity in the solid MBM residue but reduced infectivity by approximately six log orders (166+/−13 days).

No animals inoculated with biodiesel or glycerol fractions derived from infected MBM (Biodiesel sc, Glycerol sc) died with scrapie symptoms. The 28% loss of animals in these groups is comparable to previous reported attrition during long-term experiments. Secondary passage of brain homogenate from these animals was free of scrapie infectivity (survival>200 days). A minor loss of animals in the negative control groups was not due to scrapie infectivity, but rather to traumatic injuries from fighting (Table S2).

#### Endpoint Western analysis

**Figure 4:**
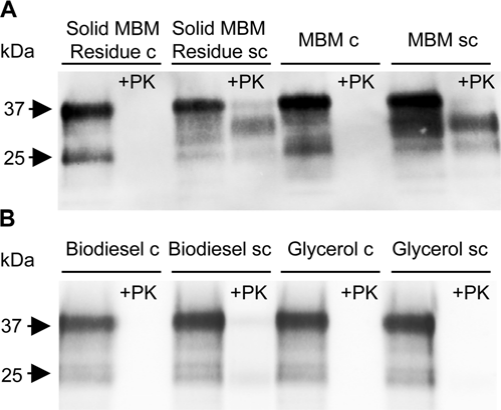
Western Blot analysis of hamster brains after termination of the infectivity bioassay. After the animal bioassay was terminated we evaluated brains from the different treatment groups for presence of PrP^sc^. We could detect PrP^sc^ in brains from animals treated with infected solid MBM residue (4A, Solid Residue sc, +PK) as well as animals that received MBM spiked with infected brain (4A, MBM sc, +PK). Samples were digested with proteinase K to verify presence of PrP^sc^ and detected with monoclonal (mAb) IPC1. Brain homogenates from inoculated biodiesel and glycerol fractions contained no PK resistant material whether derived from infected or uninfected MBM (4B). This suggests that death of animals inoculated with the solid MBM residue was definitely due to infection with scrapie whereas the biodiesel and glycerol phase of the reaction can be considered as decontaminated due to lack of any detectable protease resistant material in brains from animals injected with the respective phases.

After termination of the animal bioassay we performed Western Blot analysis of select brains from animals from the different treatment groups to determine the presence of proteinase K (PK) resistant material (Figure 4). In support of the bioassay we were able to detect PrP^sc^ in brain homogenates prepared from animals that had been inoculated with the Solid MBM Residue sc, despite the absence of detectable PrP^sc^ in the inoculum. Brains from animals inoculated with untreated MBM sc (positive control) also contained PK resistant material (Figure 4A). PrP^sc^ was undetectable in Biodiesel sc or Glycerol sc by Western Blot or bioassay (Figure 4B). None of the uninfected negative controls showed protease resistant PrP, as expected (Figure 4A and B).

These data show that the production of biodiesel and glycerol from prion contaminated MBM led to no detectable PrP^sc^ or prion infectivity in the applied assays. In contrast, the solid MBM residue retained infectivity in the animal bioassay and cannot be considered as decontaminated and thus could still serve as a vector for disease transmission.

### Brains from animals inoculated with solid MBM residue show typical spongiform degeneration and vacuolation

**Figure 5:**
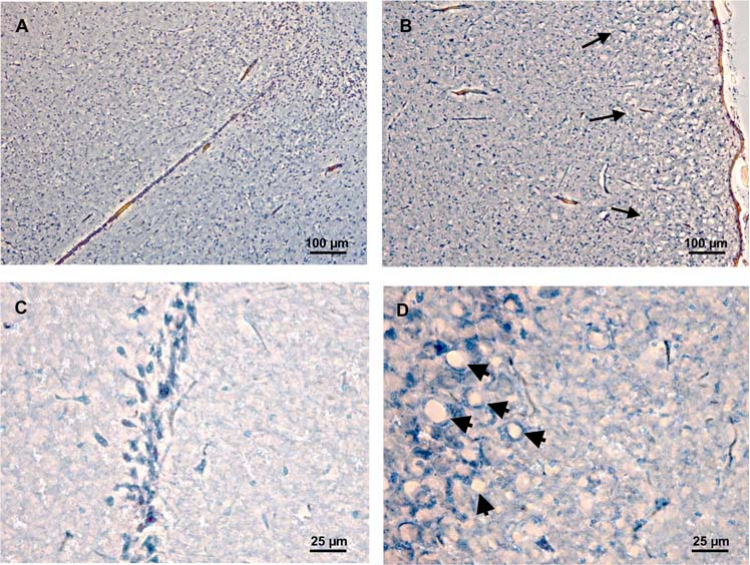
Histochemistry of coronal sections from brain of hamsters treated with solid MBM residue. Coronal brain sections were stained with Hematoxylin-Congo red. (A, B) Control animals inoculated with the solid MBM residue resulting from the reaction with MBM+5% uninfected brain exhibited normal brain morphology without vacuolation or amyloidosis. (C, D) Cryo-sections of hamster brain stained with Hematoxylin-Congo red show vacuolation (arrows) when animals were inoculated with the Solid MBM residue resulting from the reaction with MBM+5% infected brain suggesting scrapie infection. Both animals were sacrificed 150 days post-inoculation, when the infected animal displayed severe scrapie symptoms.

To confirm scrapie brain pathology from animals with clinical scrapie symptoms we evaluated brain sections counterstained with Hematoxylin-Congo red. Typical vacuolation of prion disease was observed in brain sections from animals inoculated with the Solid MBM Residue sc (Figure 5C and D). Control animals inoculated with the Solid MBM residue resulting from the reaction with MBM+5% uninfected brain (Solid MBM Residue c) exhibited normal brain morphology without vacuolation (Figure 5A and B).

### Biodiesel process does not change the specific infectivity of the prion protein

Secondary passage of brain homogenates was conducted from animals in all reaction groups. Brain homogenates from animals that received Solid MBM Residue sc, MBM sc and Biodiesel sc and Glycerol sc were inoculated into new groups of hamsters to assess results from the initial bioassay. Animals that received inocula prepared from brains that were treated with Solid MBM Residue sc and MBM sc developed clinical scrapie symptoms at 70 days and were sacrificed at 80 days (data not shown). This confirms that MBM sc can transmit scrapie following alkaline methanolysis. Survival time after re-inoculation was comparable to the survival time after inoculation of a standard 1% scrapie brain homogenate (Figure 3A). These data suggest that the biodiesel process did not change the specific infectivity of the prion protein. Animals that received inocula prepared from Biodiesel sc or Glycerol sc brains did not exhibit scrapie symptoms and survived at least 200 days, confirming that the biodiesel and glycerol phase showed no detectable prion protein infectivity after the process.

## Discussion

Decontamination of pathogenic prions has turned out to be a challenging endeavor. Prions are known to be unusually resistant to common decontamination methods. BSE is believed to be a result of insufficient decontamination and rendering methods of ruminant coproducts that were used as animal feed. Although this led to a devastating feed-borne epidemic among cattle, a major concern here is the overwhelming evidence for the zoonotic transmission of bovine prions to humans [20]. Total elimination of TSEs requires methods that completely destroy any potential prion infectivity in a large scale format. Production of biodiesel from bovine fat and brain tissue has been proposed to be a useful tool for decontamination of prions resulting in safe biodiesel [21]. In our study we evaluated an inexpensive large scale method (*in situ* transesterification) for production of biodiesel for TSE decontamination potential. Furthermore we investigated potential infectivity present not only in the biodiesel but also in the two other phases developed from the process, a solid MBM residue and glycerol. The solid MBM residue is of particular interest for its potential as a nutritious feed additive for ruminants such as cattle. In our hands, under optimal conditions for transesterification, the solid MBM residue retained 7% of the initial triglyceride and 90% of the initial protein content [17]

The alkaline methanolysis method efficiently produced biodiesel from MBM spiked with hamster brain and the method eliminated PrP^sc^ detection in all products as determined by Western blot. Our biochemical results are comparable to previous studies, at least with regards to the biodiesel and glycerol phase [15]. Biodiesel and glycerol products had no detectable infectivity in our long term animal assay (survival>200d). In contrast to the biodiesel and glycerol phase, we show that the remaining solid MBM residue that had been spiked with scrapie brain retained infectivity in our sensitive bioassay. All animals inoculated with the infected solid MBM residue developed scrapie. However, increased survival time suggests the reaction did reduce infectivity in solid MBM residue from 10^−3^ ID50 to 10^−9^ ID50 (a partial decontamination of ∼6 logs), based on a standard hamster survival curve that we established in our laboratory according to previous reported results [18]. The broad distribution of time-to-death for these animals is likely due to uneven distribution of infectious material in the inoculum, as the residue produced a relatively coarse suspension in the syringe. We suggest that, in addition to disinfection by the alkaline methanolysis reaction, we observe significant partitioning of infectivity, from the liquid phases into the solid residue. Another possible explanation for increased survival of animals inoculated with the solid MBM residue could be a high binding affinity of the prion protein to MBM and thus a sustained release from MBM in the brain. A phenomenon like this was described previously for prion binding to soil minerals [22]. In our study, when spiked into MBM, PrP^sc^ was only detectable by Western Blot after boiling of sample in detergent. On the other hand we could show that control animals that received infected MBM not subjected to the reaction (MBM sc) developed disease in a time frame comparable to a standard scrapie brain homogenate.

Our results clearly show that Western Blot detection alone is insufficient to conclude on the absence of infectious prion, particularly when assessing a grossly heterogeneous sample such as MBM. This study illustrates that lack of prion detection *in vitro* does not necessarily exclude infectivity as determined by bioassay.

Furthermore the residual scrapie infectivity detected in the solid MBM residue probably limits the use of ruminant MBM as a feed additive to only non-ruminants, such as fish and fowl, as they are not susceptible to TSEs. Relatively minor variations of this reaction (e.g., more heat and/or alkali) may prove fully effective for complete destruction of infectivity in the solid MBM residue, but must be cost-effective if suspect MBM is to be considered as a ruminant feed additive.

## Materials and Methods

### GC-MS analysis

#### Sample Preparation

25 µl of the sample was dried under a constant stream of nitrogen. The dried residue was reconstituted in 1.0 ml of isooctane. 1 µl of this solution was injected into the GC-MS.

#### GC-MS Apparatus

The samples were analyzed using a Hewlett Packard HP 6890 Series GC system combined with an HP 5973 Series mass selective detector, an HP 6890 Series injector and an HP Chem Station G1701AA version A.03.00. The MS conditions were as follows: full scan mode; electron impact ionization (EI) mode: ionization energy, 70 eV; ion source temperature, 220°C; capillary direct interface heated at 260°C.

#### GC conditions

GC conditions were as follows: split less injection mode; column, HP-1MS capillary (25 m_0.25 mm I.D.), 250 nm film thickness; injection port temperature, 280°C; carrier gas, helium; flow rate, 0.6 ml/min; column temperature, 100°C increased to 310°C at 30°C/min, and was held at this temperature for 5 min.

### Source and characteristics of MBM

MBM samples were obtained through the Fats and Proteins Research Foundation (Alexandria, VA) from an anonymous member company. The supplier specified that the MBM was made entirely from cattle bones and offal. The material had about 43% protein and 37% ash.

We prepared small, representative samples from this very heterogeneous material. Initially a mortar and pestle was used to reduce the size of the largest pieces and break up large aggregates. Then the material was mixed well and split four times with a 10-slot riffle box, resulting in a ∼120 g representative sample. This material was milled under liquid nitrogen in a Spex Centriprep model 6800 mill (Spex Centiprep Inc., Metuchen, New Jersey, United States). Milling the material into such a fine powder does not represent industrial practice. This milling, however, was preferable for two reasons. First, MBM is heterogeneous at a fairly large scale; without reducing the particle size and mixing, it is unlikely that any small sample of MBM would be representative of the whole. Secondly, small particle size was a requirement for injecting the particles during the bioassay. The resulting fine powder was mixed well and split into ∼10 g samples by repeated cone-and-quartering. Samples were finally dried in a desiccator for 48 h to get rid of most of the humidity that interferes with the biodiesel reaction (in situ transesterification).

### Alkaline methanolysis

As a model for a rendered TSE-infected animal carcass we spiked 10 g MBM with 0.5 g hamster brain, froze it with liquid nitrogen, and homogenized the mixture using a mortar and pestle. Alkaline methanolysis was performed by shaking the MBM/ brain mixture vigorously in 24 ml 0.25 M sodium methoxide at 35°C for 2 hours. The study sample was spiked with brain from a scrapie infected hamster (263K), and an identical sample not subjected to the reaction served as a positive control. In parallel we performed a negative control reaction using MBM spiked with brain from a normal/uninfected hamster.

This alternate method of biodiesel production does not require prior lipid extraction but has been proven to convert lipids into Fatty acid methyl esters with high efficiency [17].

### Sample preparation for intracranial inoculation (infectivity bioassay)

The in situ transesterification of lipids to FAME typically leads to 3 different reaction phases or products which are easy to separate. The solid MBM residue was air dried overnight and crushed with mortar and pestle (without liquid nitrogen) to obtain a homogeneous sample. The solid MBM residue was suspended in 320 mM sucrose to 25 µg total protein/50 µl with gentle heating for 20 min at 40°C. The resulting non-homogenous suspension was passed through a 20 gauge needle, and then a 27 gauge needle, and some solid material was lost in each passage. The biodiesel and glycerol phases were simply diluted into 320 mM sucrose to an estimated amount of 25 µg total protein/50 µl (10-3 ID50). Under anesthesia, weanling female Syrian hamsters received 50 µl of intracranial inoculum. Inocula for toxicity studies were prepared as described above but reaction phases were diluted into 320 mM Sucrose at two different concentrations (25 µg and 0.25 µg total protein/50 µl) and only non infected MBM was used.

### Western Blotting

10% Brain homogenate was prepared at room temperature by homogenizing 1 g hamster brain with a Polytron homogenizer in 10 ml MES extraction buffer (60 mM n-Octyl-glucoside, 1% Triton-X 100, 25 mM MES-HCl, pH 6.5, 150 mM NaCl; all chemicals from Sigma-Aldrich Corp., St. Louis, Missouri, United States) containing one tablet of Roche complete mini protease inhibitor (Roche Corp., Basel, Switzerland) and 1 mM phenyl-methane-sulfonyl-fluoride (Sigma-Aldrich Corp.). The homogenate was precleared at 3000 rpm for 5 min in a centrifuge (Eppendorf Corp., Hamburg, Germany). The solution was diluted 1∶10 in the above described MES buffer and aliquoted for further use. For Western Blotting aliquots were loaded on a SDS-PAGE gel at 25 µg total protein/ lane. Protein content was measured by BCA using an Eppendorf Biophotometer.

Crude reaction fractions of biodiesel and glycerol were diluted with 4x sample loading buffer (SLB, Invitrogen Corp., Carlsbad, California, United States), heated to 70°C for 10 min and directly loaded onto a precast 4–12% gradient gel (Invitrogen Corp.). The non reacted brain spiked MBM and solid MBM residue each were boiled for 10 min in sample loading buffer and supernatants were taken off and analyzed by immunoblotting. Previous prepared inocula of the respective fractions (see above) were simply diluted and subjected to Western Blot analysis.

For detection of PrP^sc^ infectious samples were digested with proteinase K solution (Roche Corp., 15 µg/µl final concentration) for 1 hour at 60°C.

Electrophoresis was performed using an XCell SureLock Mini-Cell (Invitrogen Corp.). Protein transfer was performed wet with a Mini Trans-blot Cell (Bio-Rad, Hercules, California, United States). PrP was detected using either monoclonal mAb 3F4 or monoclonal mAb IPC1 (Sigma-Aldrich Corp.). Protein bands were visualized with an anti-mouse HRP secondary antibody (Pierce Biotechnology Inc., Rockford, Illinois, United States) and enhanced chemiluminescent detection (Pierce Biotechnology Inc.).

### Histochemistry

Hamster brains were fixed in 10% neutral buffered formalin for 48 h then sunk in 30% sucrose. Coronal cryosections (5 µm) were serially collected, attached to glass slides, counterstained with Meyer's hematoxylin and Congo Red. Sections were evaluated on an inverted Leica DMI4000B microscope and digital images collected using an attached Leica DFC320 camera.

Cryo-sections were prepared from animals sacrificed 150 days post-inoculation, when the infected animal displayed severe scrapie symptoms.

## Supporting Information

Figure S1 Acute toxicity studies. To evaluate the acute toxicity and tolerable dose of the products of the biodiesel process, we performed Western blot and intracranial inoculation in hamsters using the reaction products from uninfected brain, after dilution of the 3 product phases (25 µg and 2.5 µg protein in 50 µl respectively). Western blot analysis (upper phase (Biodiesel c) lanes 2 and 3; interphase (Glycerol c), lanes 4 and 5; and lower phase (Solid MBM Residue c), lanes 6 and 7) failed to detect PrP in any inoculum. MBM spiked with 5% brain (MBM c, lanes 8-10, 25 µg, 2.5 µg and 0.25 µg in 50 µl respectively) and uninfected/normal brain homogenate (1% NBH, lane 1) served as controls. Coomassie stain (data not shown) showed a smear of protein present in all fractions except the upper phase (Biodiesel c), indicating the presence of proteinaceous material.
(0.20 MB TIF)

Table S1 (0.03 MB DOC)

Table S2 (0.03 MB DOC)
