# Supplementary figures and images for: Prion Infected Meat-and-Bone Meal Is Still Infectious after Biodiesel Production
Source: PLoS One. 2008 Aug 13;3(8):e2969. doi: 10.1371/journal.pone.0002969 (PMC2493038; doi:10.1371/journal.pone.0002969)

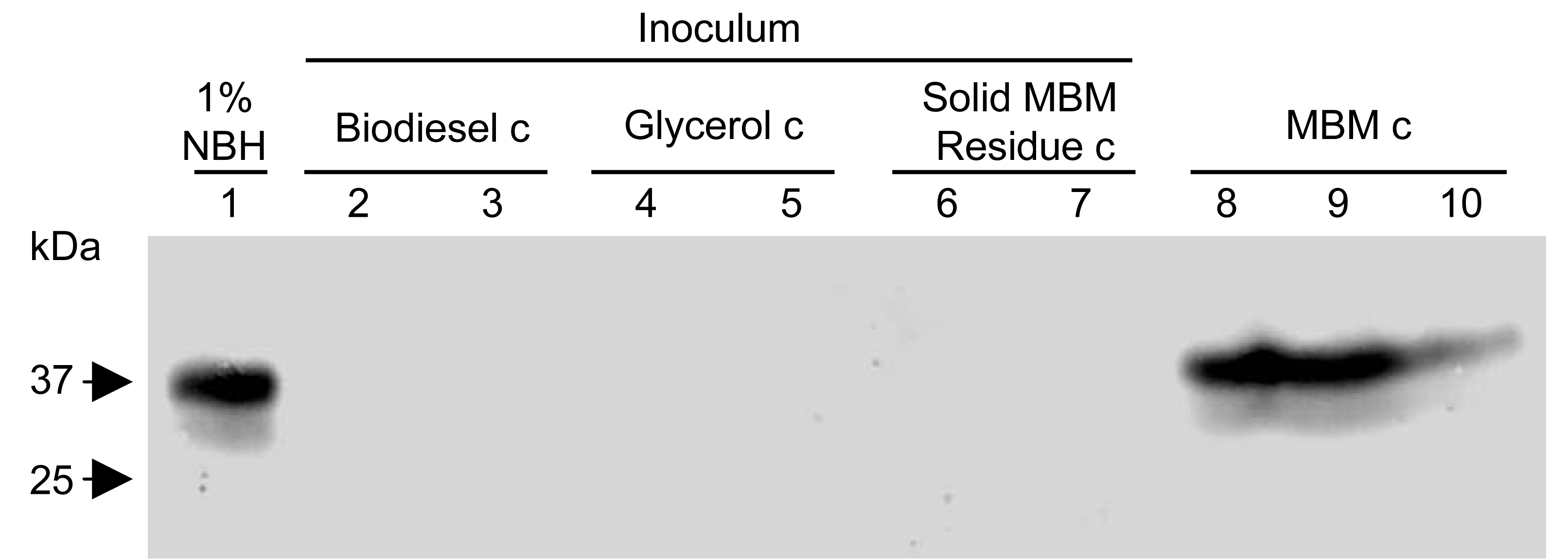

Supplement: Figure S1 — Acute toxicity studies. To evaluate the acute toxicity and tolerable dose of the products of the biodiesel process, we performed Western blot and intracranial inoculation in hamsters using the reaction products from uninfected brain, after dilution of the 3 product phases (25 µg and 2.5 µg protein in 50 µl respectively). Western blot analysis (upper phase (Biodiesel c) lanes 2 and 3; interphase (Glycerol c), lanes 4 and 5; and lower phase (Solid MBM Residue c), lanes 6 and 7) failed to detect PrP in any inoculum. MBM spiked with 5% brain (MBM c, lanes 8-10, 25 µg, 2.5 µg and 0.25 µg in 50 µl respectively) and uninfected/normal brain homogenate (1% NBH, lane 1) served as controls. Coomassie stain (data not shown) showed a smear of protein present in all fractions except the upper phase (Biodiesel c), indicating the presence of proteinaceous material. (0.20 MB TIF) [file pone.0002969.s001.tif]
