# Supplementary material for: Prion Infected Meat-and-Bone Meal Is Still Infectious after Biodiesel Production
Source: PLoS One. 2008 Aug 13;3(8):e2969. doi: 10.1371/journal.pone.0002969 (PMC2493038; doi:10.1371/journal.pone.0002969)
Supplement: Table S1 — (0.03 MB DOC) [file pone.0002969.s002.doc]

| **Inoculum**  **(uninfected)** | **Animals Alive/**  **Total Animals 24 hours** | **Animals Alive/**  **Total Animals 90 days** |
| --- | --- | --- |
| Biodiesel high dose* | 5 | 5 |
| Biodiesel low dose** | 5 | 5 |
| Glycerol high dose | 4 | 4 |
| Glycerol low dose | 4 | 4 |
| Solid MBM Residue high dose | 6 | 6 |
| Solid MBM Residue low dose | 6 | 6 |

**Table S1.** Acute toxicity studies: all animals survived at least 90 days, when study

was terminated

* High dose represents a dilution of 1:1K in 320mM sucrose solution. This is comparable to a hypothetically brain equivalent protein content of 2.5µg/ 50 µl inoculum based on the input of total brain in the reaction mixture.

** Low dose represents a dilution of 1:10K in 320mM sucrose solution. This is comparable to a hypothetically brain equivalent content of 25µg/ 50 µl inoculum based on the input of total brain in the reaction mixture.
