# Supplementary material for: Prion Infected Meat-and-Bone Meal Is Still Infectious after Biodiesel Production
Source: PLoS One. 2008 Aug 13;3(8):e2969. doi: 10.1371/journal.pone.0002969 (PMC2493038; doi:10.1371/journal.pone.0002969)
Supplement: Table S2 — (0.03 MB DOC) [file pone.0002969.s003.doc]

**Table S2.** All control animals

survived at least 180 days and did not exhibit clinical scrapie symptoms*

| **Inoculum** | **spiked with**  **Uninfected Brain**  **(Sacrificed Animals/ Total animals)** | **Day Sacrificed**** |
| --- | --- | --- |
| Biodiesel phase | 0/10 | **>200** |
| Glycerol phase | 2/10 | **>200** |
| Solid MBM Residue  + 5% brain | 3/10 | **>200** |
| MBM  + 5% brain | 1/6 | **180** |
| Brain Homogenate 1% | 2/10 | **>200** |

* intercurrent death were most likely due to injuries

occurring during long term caging of animals

** study was terminated at this point
